# Modification of Structural and Photocatalytic Properties of Pure and Vanadium-Doped Sol–Gel Zinc Oxide Films by Adding Graphene Oxide Dispersion

**DOI:** 10.3390/nano16140888

**Published:** 2026-07-19

**Authors:** Igor A. Pronin, Alexander S. Kitaev, Ivan A. Filippov, Alexey S. Komolov, Andrey A. Karmanov, Nadezhda D. Yakushova, Vitalii A. Solov’ev, Ghenadii Korotcenkov

**Affiliations:** 1 Department of Nano- and Microelectronics, Penza State University, 440026 Penza, Russia; ar7style436@gmail.com (A.S.K.); iffilippoff@yandex.ru (I.A.F.); starosta07km1@gmail.com (A.A.K.); yand93@mail.ru (N.D.Y.); vitas_psu@mail.ru (V.A.S.); 2 Department of Physics, St. Petersburg State University, 199034 Saint Petersburg, Russia; 3 Department of Physics and Engineering, Moldova State University, 2009 Chisinau, Moldova; ghkoro@yahoo.com

**Keywords:** zinc oxide, photocatalyst, graphene oxide, selective phase separation, sol–gel technology

## Abstract

The work explores the effect of modifying thin ZnO and ZnO:V sol–gel films with graphene oxide (GO) dispersions on their structural and photocatalytic properties. The study has, for the first time, detected the effect of selective phase separation in sols, characterized by the separate crystallization of zinc and vanadium oxides upon adding GO dispersion into a mixed sol. Increasing the GO concentration in ZnO-VO_2_ precursor sols improves the crystallinity of the material; films of the same composition without added GO are X-ray amorphous. Conversely, adding GO to unmodified ZnO sols causes a reduction in the crystallite size of the films, which increases with higher GO content. Notably, their photocatalytic activity varies non-monotonically: at 10 wt.% GO, it is minimal, while a further increase in the GO concentration leads to its improvement. An increase in the GO concentration in ZnO:V films causes a monotonically enhanced efficiency of photocatalysis. This may be related to the improved crystallinity and the formation of a percolation cluster from reduced graphene oxide.

## 1. Introduction

**Figure 1:**
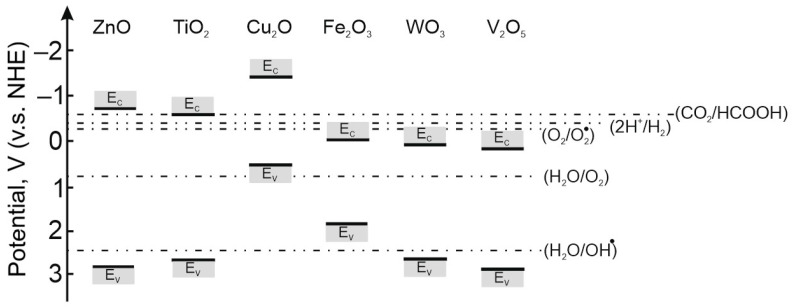
Band structure of photocatalysts based on transition element oxides.

Nowadays, semiconductor photocatalysts are promising materials in alternative power engineering in terms of generating renewable energy sources and cleaning the environment from various pollutants [1,2]. Traditional photocatalysts such as zinc oxide, titanium oxide, tungsten oxide and others act as wide-band gap semiconductors [3]. Figure 1 illustrates the band structure characteristics of transition metal oxide photocatalysts [4,5,6]. It is evident that this class of materials is prospective not only for the removal of pollutants from wastewater but also for water photolysis and the conversion of carbon dioxide into organic molecules.

Zinc oxide is particularly notable due to its high charge carrier mobility, optimal band gap structure and manufacturability [7,8]. In particular, the energy position of the conduction band minimum and valence band maximum enables the photolysis of water, transformation of carbon dioxide into organic molecules, and purification of water and air from organic pollutants [7,8]. A major limitation of pure semiconductor photocatalysts, including ZnO, is their ability to operate effectively only under ultraviolet (UV) radiation. At Δ*E_g_* = 3.37 eV for ZnO [9], band gap absorption is possible only at a wavelength shorter than 368 nm. This significantly limits the use of sunlight as a renewable energy source for the excitation of photocatalysts. As is known, ultraviolet radiation makes up only 3–7% of the total solar radiation reaching the Earth’s surface [10]. Traditional methods to solve this problem involve broadening the absorption spectrum by modifying pure semiconductors with impurity atoms (metallic and non-metallic elements) [11,12]. At low impurity concentrations, a substitutional solid solution typically forms; at higher concentrations, the synthesis of heterogeneous systems is possible [13].

However, traditional approaches do not always enhance the efficiency of photocatalytic materials [14]. In this regard, the search for new approaches and new materials to modify the photocatalysts is particularly relevant. Experiments have shown that such new materials can be two-dimensional materials such as graphene [15], graphene oxide [16], and carbon nitride [17], which have aroused significant interest in the last decade. Graphene oxide (GO) holds a special place among 2D materials due to its semiconducting properties, high electrical conductivity, a wide range of adsorption centers on the surface, and ease of addition as dispersions into the solution used for the wet chemical synthesis of semiconductor oxides [18]. Nevertheless, this material has not been sufficiently examined in terms of modifying the photocatalytic properties of semiconductor oxides. This work reports a new effect of selective phase separation initiated by adding graphene oxide dispersions to mixed precursor sols of zinc and vanadium oxides. Based on this effect, it is possible to synthesize composite materials with a controlled band gap structure that demonstrate improved photocatalytic properties.

## 2. Materials and Methods

### 2.1. Synthesis of Samples

ZnO films were generated by the sol–gel method using the following compounds: zinc acetate dihydrate, 2-methoxyethanol and 2-aminoethanol (Sigma-Aldrich, St. Louis, MO, USA). The substances were mixed in a flask for 20 min, after which the resulting sol was matured for 1 h at 60 °C and 24 h at room temperature (21 °C). The sol films were applied to the silicon substrates by spin-coating and annealed for 1 h at 350 °C in air. To get ZnO:V (1, 3, 5 at.%) films, ammonium metavanadate (Sigma-Aldrich, St. Louis, MO, USA) was added to the initial solution, and the substances were synthesized according to the same scheme. To modify ZnO and ZnO:0.05V films with graphene oxide (AkKo Lab, Moscow, Russia), dispersions of graphene oxide in 2-methoxyethanol were prepared and added to the initial sols at concentrations of 10, 20, and 30 wt.% in terms of the mass of the oxide films generated by annealing (ZnO-GO (10 wt.%), ZnO-GO (20 wt.%), ZnO-GO (30 wt.%), ZnO:0.05V-GO (10 wt.%), ZnO:0.05V-GO (20 wt.%), ZnO:0.05V-GO (30 wt.%) samples). To improve the homogeneity of GO distribution in the sol, the resulting solutions were ultrasonicated. Similarly to the ZnO films, all generated films were annealed for 1 h at 350 °C in air. The average film thickness was determined to be (1.5 ± 0.4) µm using scanning electron microscopy on a cross-sectional specimen.

### 2.2. Sample Characterization Techniques

The X-ray diffraction (XRD) measurements were carried out using the D8 Discover apparatus (Bruker, Billerica, MA, USA) equipped with the Cu Kα, 0.15406 nm wavelength X-ray excitation source. The film samples were subjected to the measurements in the theta/2 theta geometry.

The surface structure of the samples was studied using the scanning electron microscope VEGA 3 SBH (TESCAN, Brno, Czech Republic) with the reflection electron detector.

The chemical composition of the surface of the obtained samples was analyzed using X-ray photoelectron spectroscopy. The XPS spectra were measured under UHV conditions (10^−7^ Pa) using the Escalab 250Xi X-ray photoelectron spectrometer (Thermo Fisher Scientific Inc., Waltham, MA, USA) with photon energy Al-Kα = 1486 eV. The XPS peak deconvolution was carried out by Shirley background subtraction followed by peak fitting to the Voigt functions having a mixed Gaussian and Lorentzian character. The surfaces under study were sputtered using an Ar^+^ ion beam at 500 eV beam energy for 30 s.

The transmission spectra of the synthesized samples were examined using the SF-56 spectrophotometer (LOMO, Saint Petersburg, Russia). The transmission spectra were converted into absorption spectra in Tauc coordinates according to the method described in the publication [19].

The photocatalytic properties of all generated films were examined in model reactions of methyl orange decomposition. The films were formed on a 76 mm × 26 mm glass substrate and placed in Petri dishes. The initial dye concentration in all experiments was 5 ppm, and the dye volume was 60 mL in all cases. During all photocatalytic experiments, the aqueous methyl orange solution was stirred magnetically at 400 rpm. The DB 8 low-pressure linear mercury lamp (LISMA, Saransk, Russia) was employed as a radiation source; the lamp spectrum included the following most intense lines: 254 nm (4.9 eV); 405 nm (3.06 eV); 436 nm (2.84 eV); 546 nm (2.2 eV). The irradiation power was 8 W. The lamp was positioned collinearly with the samples under study at a distance of 7 cm from the film surface, ensuring uniform illumination intensity. During the experiment, 3 mL samples were taken from the solution every 30 min, after which the absorption coefficient was spectrophotometrically measured at a wavelength of 465 nm, corresponding to the maximum absorption of the methyl orange solution [20], and its concentration was recalculated according to the Bouguer–Beer–Lambert law [21]. Spectrophotometric measurements were performed in triplicate at each stage of the photocatalytic process to assess the uncertainty in concentration determination. After each measurement, the samples were returned to the solution, ensuring a constant volume throughout the experiment. To prevent the decreasing concentration of the dye in the solution for reasons not related to photocatalysis (adsorption of molecules on the film; direct decomposition in the solution upon exposure to UV radiation), we concurrently ran controlled experiments, the results of which are taken into account in the final calculation of the concentration [22].

First, the samples were exposed to the pollutant solution for 30 min prior to irradiation to ensure adsorption-desorption equilibrium. The initial time point and the initial concentration (C_0_) were chosen right after that. Second, measurements of potential degradation or concentration changes of methyl orange were conducted under the conditions of UV irradiation and in the absence of a photocatalyst. The corresponding results are presented in the plots of dye concentration versus time.

## 3. Results

### 3.1. Modification of ZnO Films with Graphene Oxide Suspension

**Figure 2:**
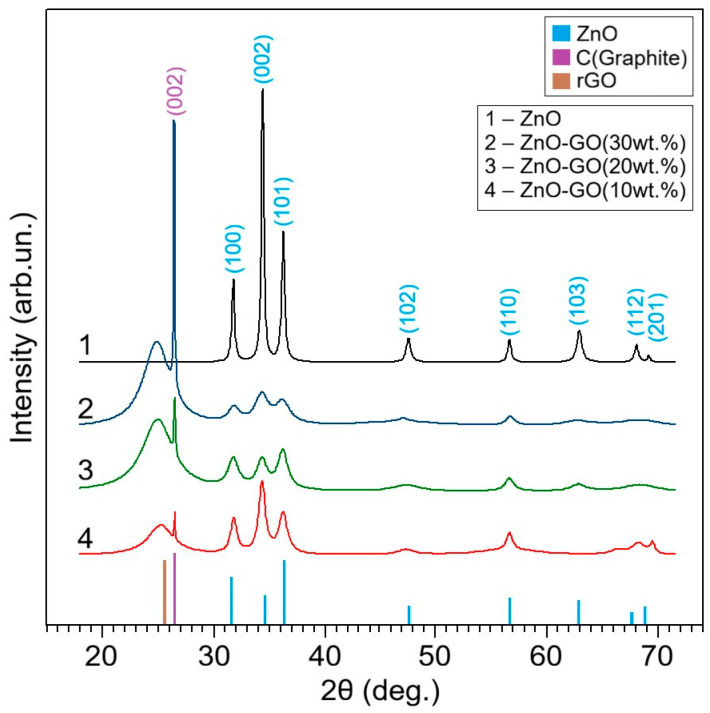
Diffraction patterns of ZnO films modified with GO (1—ZnO; 2—ZnO-GO (30 wt.%); 3—ZnO-GO (20 wt.%); 4—ZnO-GO (10 wt.%)).

We consider the effects of adding graphene oxide as a suspension to ZnO films at the stage of sol generation on the phase composition of the samples. Figure 2 shows the diffraction patterns of pure and GO-modified films. Pure ZnO films exhibit only reflections characteristic of the wurtzite-type phase (JCPDS 36-1451 [23]) and feature misoriented crystallites. Their average size *d* was calculated for the three most intense reflections ((100), (002), (101)) using the Scherrer equation [24]:

(1) $$d=\frac{\kappa \lambda }{\beta \cdot \cos\left(\Theta \right)},$$

where κ is the crystallite shape factor (without data on the shape, κ = 0.9 is a good approximation), λ is the X-ray wavelength (1.54 Å for Cu *K*α), and β is the full width of the reflection at half maximum; Θ is the Bragg angle. As an example, Table 1 presents data calculated for a pure ZnO film, based on the approach of [25].

The calculated value was *d* = 30 nm (given in Table 1), which is a typical value for ZnO films derived by the sol–gel method [26].

Adding graphene oxide into the system at (10–30) wt.% concentrations changes the diffraction pattern (Figure 2). The reflections corresponding to the ZnO phase primarily broaden and decrease in intensity, indicating a reduction in the crystallite size of the material with higher GO concentration. In addition, a reflection corresponding to the family of planes (002) of graphite (2Θ = 26.5°) and a wide reflection (22° < 2θ < 28°) corresponding to reduced graphene oxide (*r*GO) appear [27]. The appearance of these phases is linked to the processes of thermal reduction of graphene oxide, during which the groups adsorbed on its surface (-COOH, -OH, H_2_O and others) are removed, reducing the distance between the carbon sheets. It should be noted that the increase in GO concentration within the film is accompanied by a shift of the broad reflection (22° < 2θ < 28°) toward lower angles. This indicates an increase in the interlayer spacing in *r*GO, which is apparently due to the slower removal of surface groups in films containing a higher proportion of *r*GO. The reflections corresponding to graphite and *r*GO intensify with the content of graphene oxide added to the ZnO film.

Table 2 presents the values of the average sizes of ZnO nanocrystallites in the studied films, calculated using the Scherrer equation. Apparently, the average crystallite size decreases from 11 nm to 5 nm with the increasing GO concentration from 10 wt.% to 30 wt.%. This phenomenon can be explained by several reasons: ZnO nanoparticles growing in the sol are immobilized on the functional groups of GO, and they do not engage in further agglomeration processes [28]; numerous oxygen-containing groups on the GO surface act as growth centers for ZnO nuclei, which leads to the formation of smaller particles [29]; and GO sheets distributed in the sol limit the agglomeration of ZnO particles [30].

**Figure 3:**
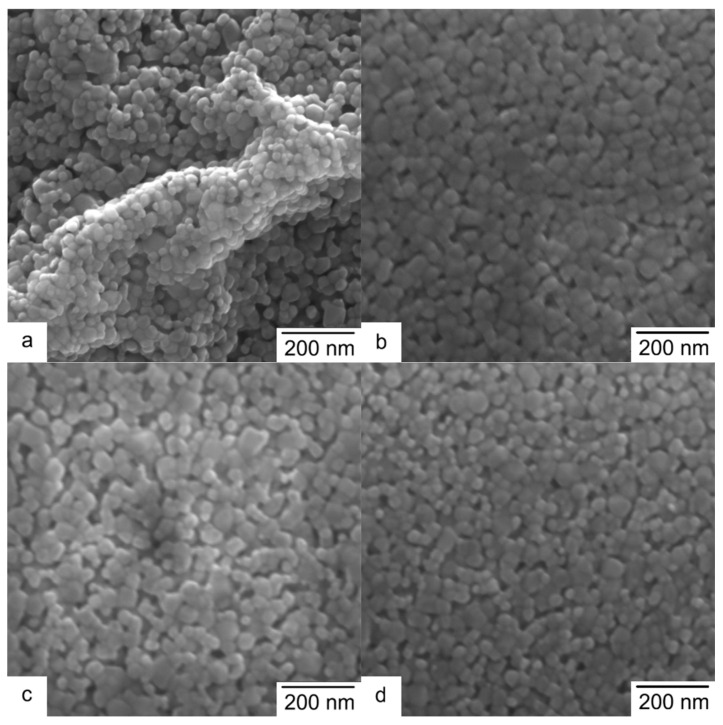
Micrographs of the samples: (**a**) ZnO; (**b**) ZnO-GO (10 wt.%); (**c**) ZnO-GO (20 wt.%); (**d**) ZnO-GO (30 wt.%).

Figure 3 shows the typical micrographs of the samples. Pure ZnO film is represented by branches assembled from grains that have a shape close to spherical. Their average size is close to the value *d* calculated from the diffraction pattern (~30 nm), which indicates that the grains are single nanocrystallites. This structure is typical of metal oxide films derived from the sol–gel method and is associated with the processes of material assembly through the cluster-to-cluster mechanism from particles formed according to diffusion-limited aggregation [31,32]. The micrographs of ZnO films containing 10, 20, and 30 wt.% GO are virtually indistinguishable from each other (Figure 3b–d) and devoid of a branched structure. The absence of branches indicates limited cluster-to-cluster aggregation due to the presence of GO nanosheets in the sol, which restricts this process. Furthermore, the ZnO phase grows on the GO surface, ultimately resulting in a less rough material after annealing compared to unmodified ZnO films. It should also be noted that the average particle sizes observed in the micrographs are larger than the nanocrystallite sizes calculated from the diffraction patterns, indicating their polycrystalline structure.

**Figure 4:**
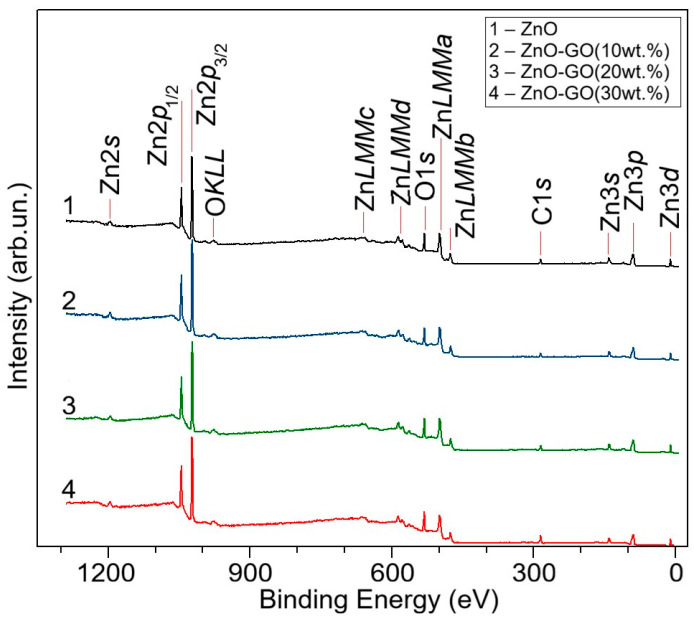
XPS survey spectra of ZnO and Zn-CO films.

Figure 4 shows the XPS spectra of the samples. It is evident that the near-surface region of the films contains Zn, O, and C.

**Figure 5:**
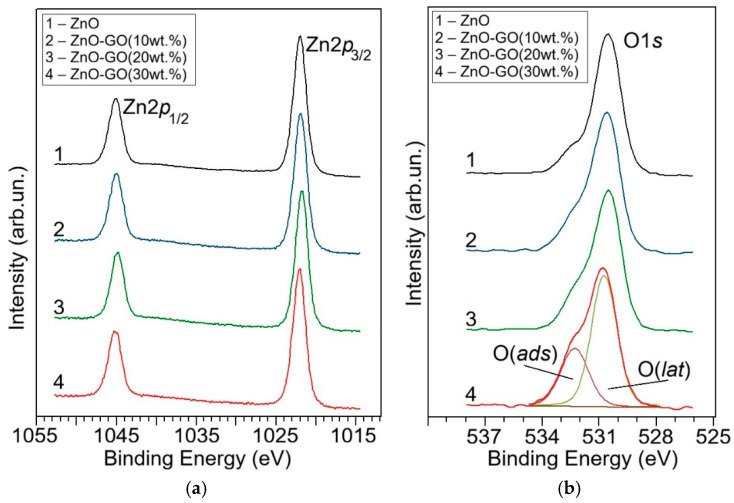
Zn2*p* (**a**) and O1*s* (**b**) spectra.

Figure 5 details the Zn2*p* and O1*s* spectra. The Zn2*p* spectrum is represented by a doublet with binding energies of ~1045 eV (Zn2*p*_1/2_) and ~1022 eV (Zn2*p*_3/2_) (Figure 5a). Since the binding energy of Zn2*p* is insensitive to the oxidation state of zinc, differentiating between ZnO and Zn(OH)_2_ is unreliable [33,34]. Conversely, the binding energy of O1*s* is very useful for this differentiation. Figure 5b shows the O1*s* spectra of the samples.

The deconvolution of the spectrum enables distinguishing two components: a low-energy one with a binding energy of ~530.5 eV and a high-energy one with a binding energy of ~532.5 eV. The first one is attributed to oxygen cations O^2−^ in the ZnO crystal lattice (O(*lat*)); the second one refers to oxygen-containing particles on the surface of the material, primarily hydroxide groups -OH (O(*ads*)) [35]. Our approach proposed in the publication [36] allows for determining the composition of the near-surface region of the films. Since the ratio is [Zn]:[O] = 1:1 in pure ZnO, the content of zinc atoms included in the oxide lattice (Zn(*lat*)) is expected to be the same in magnitude as [O(*lat*)], i.e., [Zn(*lat*)] = [O(*lat*)]. Taking this into account, the value of Zn(*lat*)/Zn indicates the content of zinc atoms found in the near-surface region and attributed to ZnO. The remaining zinc atoms are included in zinc hydroxide (Zn(OH)_2_), as well as in intermediate compounds of the sol–gel process that have not undergone the final transformation into ZnO [37]. Table 2 gives the calculated values of Zn(*lat*)/Zn for the samples. Apparently, unmodified ZnO films have Zn(*lat*)/Zn = 0.62, thus indicating that most of the zinc atoms have entered the ZnO crystal lattice. Adding graphene oxide to the films leads to a reduction in the ratio of Zn(*lat*)/Zn (0.46–0.52 for 10–30 wt.% GO), which may be linked to both a smaller crystallite size and a larger area of interaction with the environment stimulating the hydroxylation of the material, as well as to the difficulty of removing organic groups during annealing caused by the presence of graphite phases and reduced graphene oxide in the material [38].

**Figure 6:**
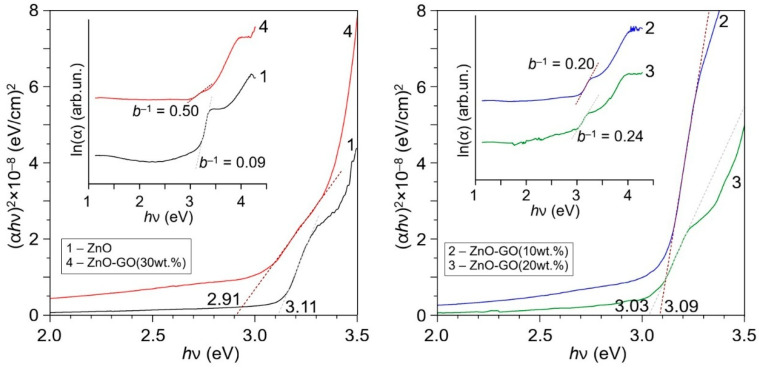
Absorption spectra of ZnO and ZnO-GO films in Tauc coordinates.

Based on the transmission spectra of the samples reconstructed in Tauc coordinates [39], the optical band gap width Δ*E_g_* and the Urbach tail energy *E_t_* were calculated. Since zinc oxide is a direct band gap semiconductor, the spectra were plotted in the coordinates (α*h*ν)^2^ = *f*(*h*ν), where α is the absorption coefficient and *h*ν is the photon energy (Figure 6), and the calculation was done using the method described in the publication [19]. However, the results obtained using this method should be treated with caution. This is primarily due to the complex phase composition of the films [40], comprising zinc oxide, GO/*r*GO, and graphite, as well as amorphous phases and hydroxylated material.

Table 2 presents the calculated values. The optical band gap of the pure ZnO sample is 3.11 eV, which is close to the value characteristic of the bulk material [41]. Enhancing the content of graphene oxide added to the ZnO sol leads to a decrease in Δ*E_g_* from 3.09 eV at 10 wt.% GO to 2.91 eV at 30 wt.% GO. Concurrently, the reduction of the band gap occurs simultaneously with a decrease in the size of nanocrystallites, which contradicts the available data: a decrease in the size of ZnO particles always leads to an increase in the energy of the optical band gap [42,43,44]. Thus, it can be assumed that the effect of decreasing the optical band gap is associated with doping ZnO with carbon [45]. Additionally, the Urbach tail energy increases from 90 meV for the pure film to 0.5 eV for ZnO-GO (30 wt.%), which is consistent with a decrease in the size of nanocrystallites. This effect is well known and is associated with greater structural disorder in small particles and lower mechanical stresses in their volume [46,47].

### 3.2. Modification of ZnO:V Films with Graphene Oxide Suspension

**Figure 7:**
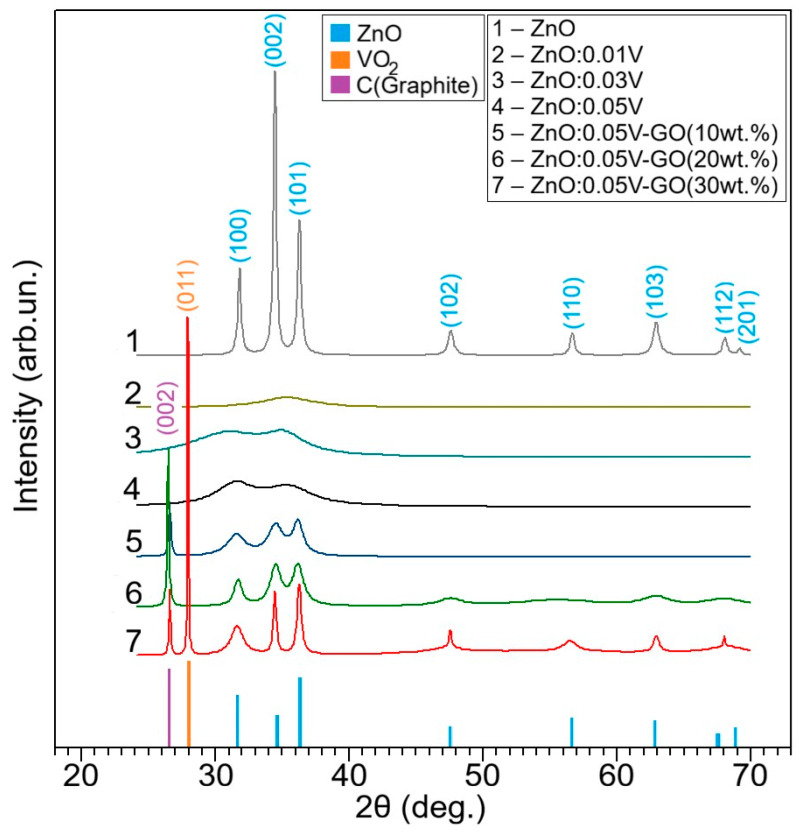
Diffraction patterns of ZnO, ZnO:V, and ZnO:V-GO films.

To modify zinc oxide films with vanadium, the concentrations of 1, 3, and 5 at.% were used. Figure 7 shows the diffraction patterns of these samples (curves 2–4). In all three cases, the material exhibits X-ray amorphous behavior. This is due to the inclusion of vanadium atoms in the zinc oxide structure, which leads to microstresses and disruptions in the crystal structure, slowing the growth of ZnO nanocrystallites to sizes smaller than 2–3 nm [48,49]. As is known, nanocrystallites of this size are X-ray amorphous [50]. Furthermore, annealing temperatures of 350 °C are no longer sufficient for the films to agglomerate into larger single crystals [51]. ZnO:0.05V samples were taken for further modification with graphene oxide.

Curves 5–7 in Figure 7 show the diffraction patterns of ZnO:0.05V films modified with 10, 20, and 30 wt.% GO, respectively (the amorphous *r*GO phase was removed from the diffraction patterns for clarity). The main features of the diffraction pattern are considered. Primarily, all films demonstrate the presence of the ZnO phase, with the larger GO mass fraction leading to the narrowing of the corresponding reflections and enhancing their intensity, which indicates the agglomeration of nanocrystallites. Calculations using the Scherrer equation showed an increase in *d* from 7 nm for ZnO:0.05V-GO (10 wt.%) to 21 nm for ZnO:0.05V-GO (30 wt.%) (the values are given in Table 3).

Notably, ZnO films without vanadium impurity show the opposite pattern: adding graphene oxide leads to a reduction in the crystallite size of the material. The second important feature is that the diffraction pattern of the ZnO:0.05V-GO (30 wt.%) sample has a narrow, high reflection corresponding to the family of (011) planes of the VO_2_ phase (2θ ≈ 28°, JCPDS 43-1051 [52,53]). All samples have a narrow reflection corresponding to the family of (002) planes of graphite (2Θ = 26.5°), formed due to the thermal reduction of GO.

**Figure 8:**
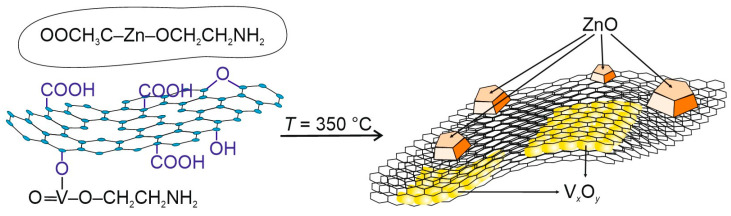
The possible model of the selective phase separation process in zinc and vanadium oxide precursor sols.

Thus, adding graphene oxide dispersions into mixed zinc and vanadium oxide precursor sols results in the phase separation of oxides in the films [54]. This effect is similar to the selective phase separation of polymers under two-dimensional confinement created by graphene oxide sheets in the solution [55]. Figure 8 schematically illustrates one of the possible models of the selective phase separation process in zinc and vanadium oxide precursor sols.

We hypothesize that the process of selective phase separation occurs during the early stages of sol aging, prior to the formation of mixed zinc–vanadium polymers. During this process, precursors containing vanadium (V) may be reduced to the vanadium (IV) state upon heating the sol due to interaction with 2-aminoethanol [56]. The resulting vanadyl cations then bind to deprotonated surface groups of the graphene oxide and become immobilized [57]. In contrast, zinc oxide precursors form sufficiently stable neutral chelate complexes within the sol [58]. These complexes interact more weakly with the graphene oxide flakes. During annealing, the oxide precursors transform into metal oxides. Specifically, vanadium oxide (V*_x_*O*_y_*) forms directly on the surface of the graphene oxide flakes, whereas zinc oxide forms within the bulk of the film. Following the aggregation of the reduced graphene oxide, the V*_x_*O*_y_* becomes encapsulated between the carbon layers, while the ZnO particles get deposited on top.

**Figure 9:**
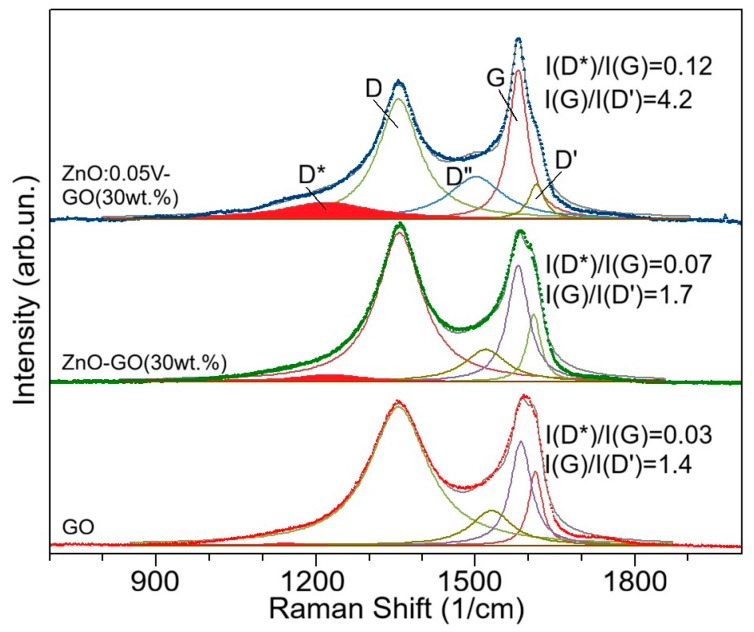
Raman spectra of the GO, ZnO-GO (30 wt.%) and ZnO:0.05V-GO (30 wt.%) films.

A GO concentration of 30 wt.% is sufficient for the almost complete removal of vanadium compounds from the sol and the generation of a sufficient amount to detect the phase by X-ray diffraction. Calculation of VO_2_ particle size using the Scherrer equation yields a value of 55 nm. The size of the ZnO nanocrystallite is 21 nm, which is close to the size of the original film (30 nm). It should be noted that the diffractometric detection of the (011) reflex does not contradict the formation of vanadium oxides (V*_x_*O*_y_*) with a different valence state. We will discuss this in greater detail during the analysis of the X-ray photoelectron spectroscopy results. The Raman spectroscopy results also support the model we developed. Figure 9 shows the Raman spectra of the graphene oxide sample deposited onto the substrate as a dispersion in 2-methoxyethanol, followed by drying for 24 h at room temperature. The results are compared with the Raman spectra from the ZnO-GO (30 wt.%) and ZnO:0.05V-GO (30 wt.%) films.

The interpretation of the results obtained was carried out according to the approach suggested in [59]. The spectra in Figure 9 are typical for GO material. The intensity ratio I(D*)/I(G) = 0.03 indicates that the GO is in an unreduced state. The ZnO-GO (30 wt.%) and ZnO:0.05V-GO (30 wt.%) samples show an increased I(D*)/I(G) ratio, which directly indicates the reduction of GO during film formation. It is also important to analyze the I(G)/I(D’) ratio, which is directly proportional to the defect concentration in the carbon framework. For the GO and ZnO-GO (30 wt.%) samples, this ratio does not differ significantly, standing at 1.4 and 1.7, respectively. However, for the ZnO:0.05V-GO (30 wt.%) films, it increases sharply to 4.2. This indicates a strong interaction between the vanadium oxide precursors and the GO surface, leading to a reduction in defect density. This is in full agreement with the concept of a possible model of selective phase separation that we have developed. Furthermore, in this series of samples, the graphene oxide is present in its most reduced state.

**Figure 10:**
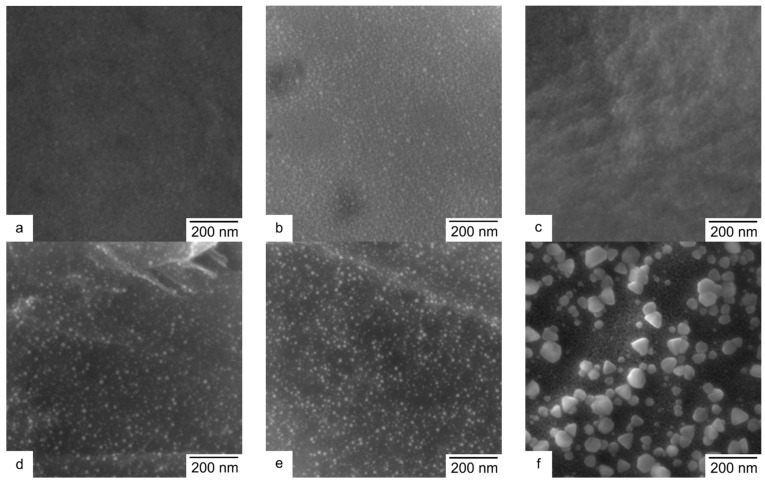
Micrographs of ZnO:V and ZnO:V-GO films: (**a**) ZnO:0.01V; (**b**) ZnO:0.03V; (**c**) ZnO:0.05V; (**d**) ZnO:0.05V-GO (10 wt.%); (**e**) ZnO:0.05V-GO (20 wt.%); (**f**) ZnO:0.05V-GO (30 wt.%).

Figure 10 shows the micrographs of ZnO:V and ZnO:V-GO films. The micrograph analysis reveals that all ZnO:V films have a similar structure with slight roughness and indistinguishable particles, consistent with the amorphous X-ray diffraction patterns of these samples. The micrographs of ZnO:V-GO films demonstrate particles, the size of which increases with the higher GO mass fraction, averaging 50 nm for ZnO:0.05V-GO (30 wt.%) samples, which is close to the size of the formed crystalline phases.

**Figure 11:**
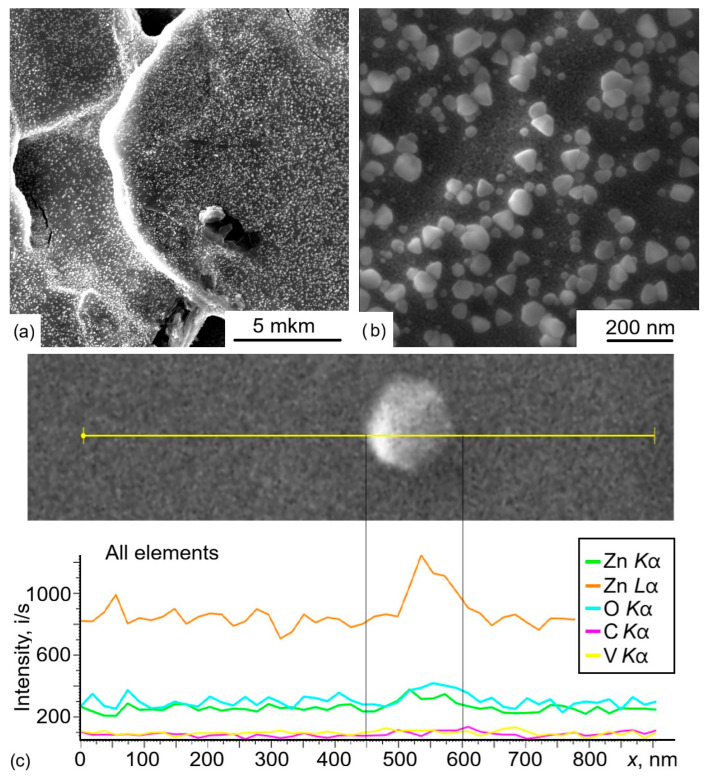
Micrographs of ZnO:0.05V-GO (30 wt.%) films in larger (**a**) and smaller (**b**) scaling; (**c**) a line-scan energy-dispersive X-ray spectroscopy of the film studied.

In order to reveal the composition of these particles, let us analyze the ZnO:0.05V-GO (30 wt.%) film in detail (Figure 11). At a large scale (Figure 11a), it can be seen that the nanoparticles are located on the surface of large carbon particles exceeding 10 µm in size. They are likely formed by a mixture of graphite and *r*GO. Line-scan energy-dispersive X-ray spectroscopy (Figure 11) revealed that those nanoscale particles are formed by zinc oxide. It should be noted that, as measured by energy-dispersive X-ray spectroscopy, the vanadium signal line proved to be much weaker than the zinc lines, which may confirm that V*_x_*O*_y_* formed within the bulk of the large carbon particles and is distributed there quite uniformly.

**Figure 12:**
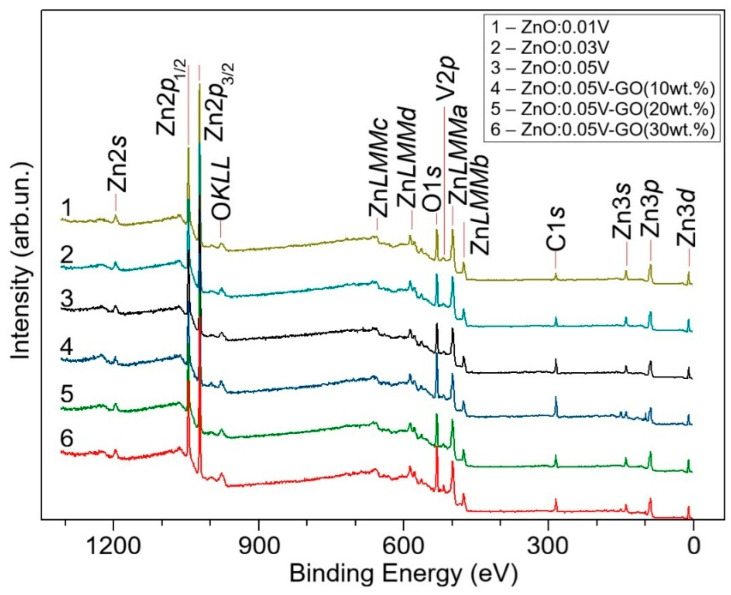
Survey XPS spectra of ZnO:V and ZnO:V-GO samples.

X-ray photoelectron spectroscopy of the films enables the calculation of their chemical composition, the valence state of vanadium in the samples, and the degree of surface hydroxylation. Figure 12 shows the overview spectra of ZnO:V and ZnO:V-GO samples. The analysis indicates that all films contain the following elements: Zn, V, O, and C. Based on these data, the [V/(V + Zn)] ratio in the films (given in Table 3) and the oxidation state of vanadium can be calculated.

Table 3 shows that in all cases the [V/(V + Zn)] ratio is close to that specified in the method at the sol preparation stage, indicating close to quantitative coprecipitation of oxides [60]. The oxidation state of vanadium in the films is very difficult to determine from the energy position of the V2*p* peak. Nevertheless, the binding energy difference Δ (E_V2*p*3/2_ − E_O1*s*_) can provide a reliable result. For all the samples under study, the Δ value is in the range of (13.3–13.7) eV. These values suggest a mixed V^5+^ and V^4^ vanadium valence state in the films. The Δ values for V^5+^ and V^4+^ reported in the literature are as follows: (12.80, 14.16) eV, (13.0, 14.40) eV, and (12.90, 13.90) eV [61], respectively. Since these data contain certain deviations, it is also necessary to examine the V2*p* core-level spectrum.

**Figure 13:**
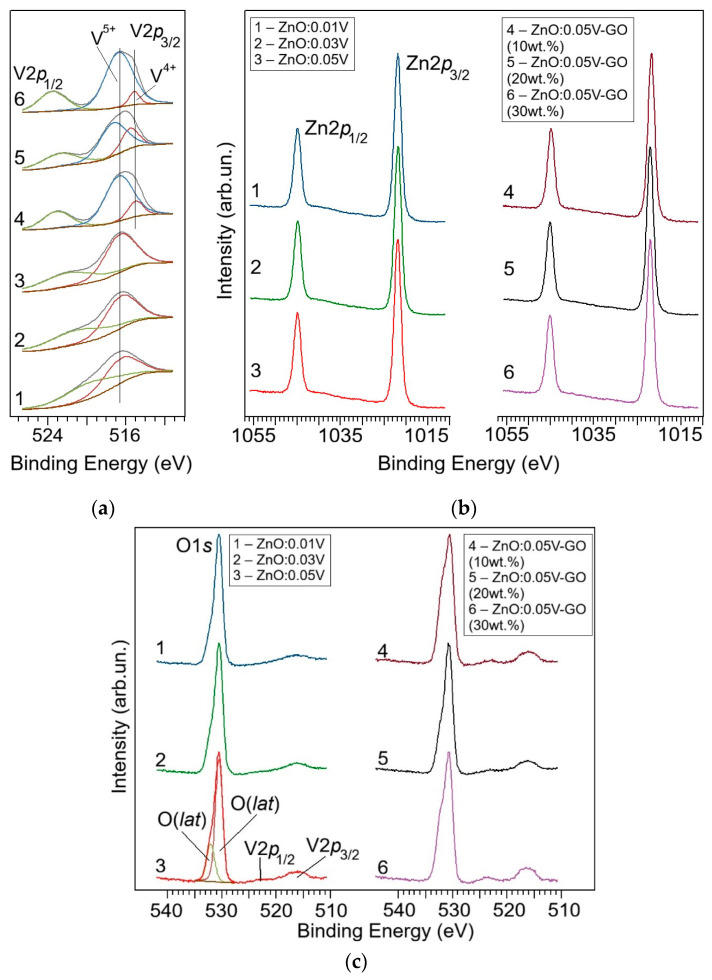
V2*p* (**a**), Zn2*p* (**b**), and O1*s* (**c**) spectra for ZnO:V and ZnO:V-GO films.

The V2*p*, Zn2*p* and O1*s* spectra of the samples are given in Figure 13. A correct interpretation of the V2*p* spectrum is a challenging task due to peak asymmetry, multiplet splitting, the “shake-up” effect, plasmon losses, argon-beam etching effects, and the overlapping of various peaks [62]. Nevertheless, by employing the approach suggested in [62], a number of important conclusions can be drawn. The spectra of all samples exhibit a V2*p*3/2 peak component with a binding energy of ~516.6 eV, which can be attributed to the V^5+^ state. Furthermore, all three graphene oxide-modified films (Figure 13, curves 4–6) exhibit a low-energy shoulder at a binding energy of ~515.2 eV, associated with vanadium in a lower oxidation state, most likely V^4+^. This is a significant observation which indicates that the presence of graphene oxide in the sols drives the reduction of vanadium from V^5+^ to V^4+^. In the case of the ZnO:0.05V-GO (30 wt.%) sample, the fraction of VO_2_ appears to be sufficient for detection by X-ray diffraction. Thus, it can be concluded that vanadium appears in the samples studied in a mixed-valence state.

Calculated Zn(*lat*)/Zn values for the films are given in Table 3. Apparently, this value for all ZnO:V samples falls within a narrow range (0.49–0.51), which is significantly lower than that for pure ZnO films (Zn(*lat*)/Zn = 0.62). This indicates a higher degree of hydroxylation of zinc atoms in the films, which correlates with their X-ray amorphousness. Adding graphene oxide to ZnO:0.05V films leads to an increase in the Zn(*lat*)/Zn ratio, which is in the range (0.52–0.55) for the films modified with (10–30) wt.% GO. In general, this also correlates with the enhanced crystallinity of these samples and the formation of larger oxide particles.

**Figure 14:**
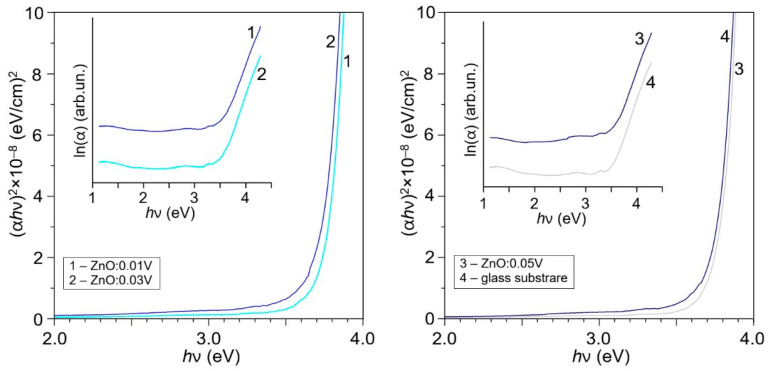
Absorption spectra of ZnO:V films in Tauc coordinates.

The absorption spectra of ZnO:V films, plotted in Tauc coordinates for all three samples (Figure 14), demonstrate the absence of the linear region characteristic of semiconducting crystalline oxides. This result is consistent with the X-ray amorphism and high degree of hydroxylation of the films obtained using XRD and XPS.

**Figure 15:**
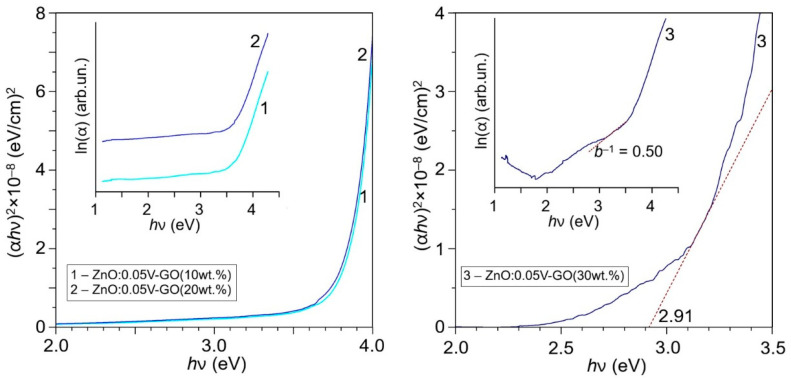
Absorption spectra of ZnO:0.05V-GO films in Tauc coordinates.

Figure 15 shows the spectra of ZnO:0.05V films modified with 10, 20 and 30 wt.% GO.

Apparently, the ZnO:0.05V-GO (10 wt.%) and ZnO:0.05V-GO (20 wt.%) samples do not exhibit the optical band gap characteristic of crystalline oxides. Thus, the ZnO-GO (10 wt.%) and ZnO-GO (20 wt.%) films, which have *d* and Zn(*lat*)/Zn values close to those of the aforementioned samples, exhibit an optical gap of (3.03–3.09) eV. This is probably due to the misorienting effect of vanadium in the ZnO crystal lattice. The GO concentration of (10–20) wt.% is insufficient for the complete separation of vanadium from the sol bulk; therefore, some of its atoms enter the zinc oxide structure.

The ZnO:0.05V-GO (30 wt.%) samples show a different pattern: a linear region can be identified in the absorption spectrum, corresponding to an optical band gap of 2.91 eV, which is consistent with the ZnO-GO composite material. Furthermore, the Urbach tail energy of this sample can be estimated at 0.50 eV. This confirms the almost complete removal of vanadium from the sol due to selective phase separation and the generation of nanocrystalline ZnO.

### 3.3. Examining Photocatalytic Decomposition of Methyl Orange on the Derived Films

**Figure 16:**
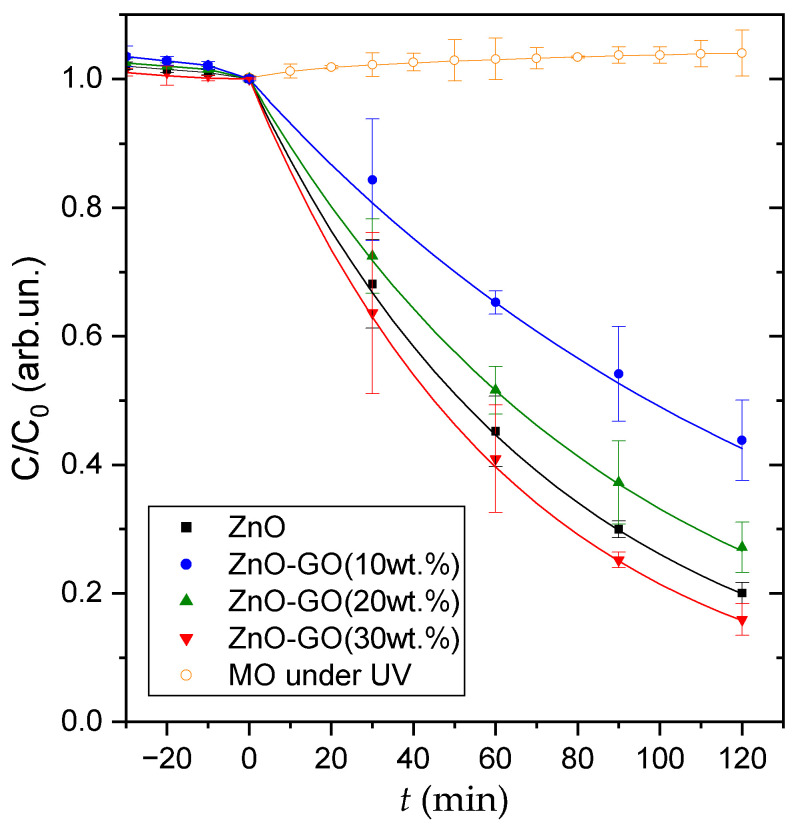
Kinetic curves of the photocatalytic decomposition of methyl orange solutions on ZnO and ZnO-GO films.

Figure 16 shows the kinetic curves for the decomposition of methyl orange solutions on ZnO and ZnO-GO films. In all cases, the dependencies are well described by an exponential curve, indicating a pseudo-first-order process [63]. The obtained data enabled the calculation of the rate constants of photocatalysis processes, which are exponent indicators (given in Table 4). However, the calculated apparent rate constants for the photocatalytic decomposition of the solution do not directly provide one with a conclusion regarding the reaction mechanism. This is because the incorporation of graphene oxide into the films leads to significant changes in the material’s morphology, crystallite size, the ratio of amorphous to crystalline phases, mass, and optical absorption. The low coefficients of determination (*R*^2^) observed for a number of samples may be attributed to these factors.

Adding graphene oxide dispersion into the ZnO film leads to a nonlinear dependence of the rate constant on the GO concentration. When adding 10 wt.% to ZnO (ZnO-GO (10 wt.%) sample), *k* decreases almost by a factor of 2 (from 3.3∙10^−3^ min^−1^ to 1.8∙10^−3^ min^−1^). A further increase in the GO content results in a higher *k*, and for the ZnO-GO (30 wt.%) sample, the constant already exceeds the values for the unmodified ZnO film. This pattern may be linked to several competing factors. As a first approximation, the efficiency of the photocatalytic material is determined by the balance between the rates of photogeneration and recombination of electron–hole pairs [64,65]. Therefore, a decreasing rate constant with enhancing Urbach tail energy should be expected. Since the localized states in the tail act as recombination centers, the recombination rate increases sharply with the enhancing Urbach energy according to a complex dependence close to exponential (more details can be found in the publication [66]). This explains the initial decrease in the *k* value upon modification of the ZnO films with 10 wt.% GO. A further increase in *k* may be linked to a number of factors that together compensate for the higher recombination rate at the tail states. This may be due to the formation of an *r*GO percolation cluster, which does not form at lower concentrations. However, the formation of a percolation cluster has not been confirmed by other methods. Therefore, this mechanism requires further research. Reduced graphene oxide acts as an electron acceptor, reducing the recombination rate, while its percolation network facilitates the rapid transport of photoinduced charge carriers through the material [67]. A reduction in ZnO nanoparticle size may also contribute to the observed phenomenon by increasing the specific contact area between the photocatalyst and the pollutant solution [68,69].

**Figure 17:**
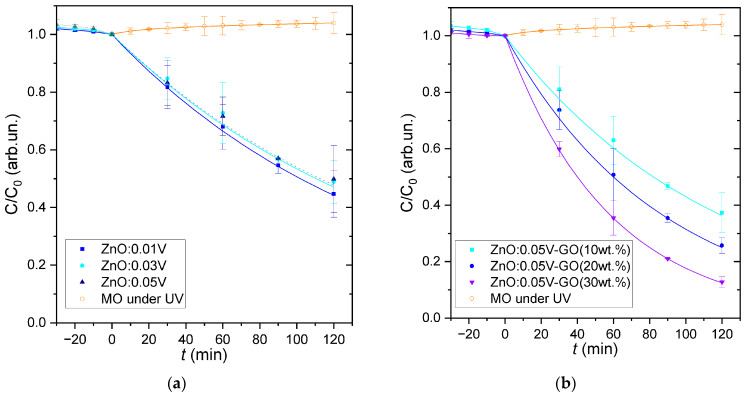
Kinetic curves of the photocatalytic decomposition of methyl orange solutions on ZnO:V (**a**) and ZnO:0.05V-GO (**b**) films.

The kinetic curves for the photocatalytic decomposition of methyl orange on vanadium-modified zinc oxide films are given in Figure 17a. Apparently, the exponential time dependence (pseudo-first-order reaction) is preserved for all three samples. Calculation of the rate constants yields values of (1.6–1.7)∙10^−3^ min^−1^, which are lower compared to ZnO and ZnO-GO.

These findings can be explained by the fact that all ZnO:V samples are X-ray amorphous. The photogeneration of electron-hole pairs occurs in a crystalline semiconductor, so the efficiency of photocatalysis generally reduces with the decreasing degree of crystallinity of the material [70,71].

Adding GO to the ZnO:0.05V system causes an increase in the *k* value (the kinetic curves for ZnO:0.05V-GO films are given in Figure 17b), and the constant also increases with the higher GO content. The maximum *k* value of 4.3∙10^−3^ min^−1^ corresponds to the ZnO:0.05V-GO (30 wt.%) sample. A number of factors contribute to this pattern. Firstly, with the higher GO content, the crystallinity of zinc oxide improves and the degree of its hydroxylation decreases. This indicates that an increasingly larger volume of the film is occupied by the crystalline semiconductor, in which charge carrier photogeneration processes occur. A significant contribution can be provided by the *r*GO percolation cluster penetrating the film, as well as by the formation of semiconducting vanadium oxide phases within the sample.

## 4. Conclusions

This work explores the effect of sol–gel modification of zinc oxide films with vanadium atoms, graphene oxide, and these components taken simultaneously on the structural and photocatalytic properties of the material. The results prove that adding graphene oxide to ZnO films at the sol generation stage leads to a reduction in the crystallite size of the material with the higher GO content. Thus, the photocatalytic activity is non-monotonic: at 10 wt.% GO, the decomposition rate constant decreases compared to pure ZnO, and then, at 20 and 30 wt.% GO, it increases. This is due to the competing factors that differently affect the efficiency of photocatalysis: enhancing the Urbach tail energy results in improving the charge carrier recombination rate, while decreasing the nanocrystallite size accounts for increasing the surface area of the material. The formation of an *r*GO percolation cluster creates pathways for the transport of photogenerated charge carriers within the material, thereby reducing the recombination rate. These processes can also improve the photocatalytic properties of the material. Adding vanadium to ZnO at a concentration of 1–5 at.% results in generating X-ray amorphous materials with inferior photocatalytic activity. This is linked to the low content of crystalline semiconductor in the films, in which the photogeneration processes occur. Adding graphene oxide dispersions to ZnO:0.05V leads to an unexpected effect of selective phase separation. Moreover, with the higher GO concentration, the crystallinity of the material improves (the opposite is observed for unmodified ZnO samples). Simultaneously, the photocatalytic activity of the samples enhances.

## Figures and Tables

**Table 1:** Data for calculating *d* of ZnO film.

Reflection	2 Θ, °	Θ, °	cos(Θ)	β, 10^−3^, rad	*d_i_*, nm	*d*, nm
(100)	31.79	15.90	0.962	2.62	28	30
(002)	34.43	17.22	0.955	2.09	35	
(101)	36.27	18.14	0.950	2.80	27	

**Table 2:** Parameters of ZnO and ZnO-GO samples estimated by XRD, XPS and optical measurements.

Sample	*d*, nm (XRD)	Zn(lat)/Zn (XPS)	ΔE_g_, eV	E_t_, eV
ZnO	30	0.62	3.11	0.09
ZnO-GO (10 wt.%)	11	0.48	3.09	0.20
ZnO-GO (20 wt.%)	7	0.52	3.03	0.24
ZnO-GO (30 wt.%)	5	0.46	2.91	0.50

**Table 3:** Parameters of ZnO:V and ZnO:V-GO samples.

Sample	*d*, nm (XRD)	V/(Zn + V), ·100%	Δ (E_V2*p*3/2_, E_O1*s*_), eV	Zn(*lat*)/Zn (XPS)	Δ*E_g_*, eV	*E_t_*, eV
ZnO:0.01V	<2	1.3	13.3	0.49	-	-
ZnO:0.03V	<2	2.5	13.7	0.49	-	-
ZnO:0.05V	<2	4.4	13.6	0.51	-	-
ZnO:0.05V-GO (10 wt.%)	7	5.8	13.3	0.55	-	-
ZnO:0.05V-GO (20 wt.%)	9	4.9	13.4	0.52	-	-
ZnO:0.05V-GO (30 wt.%)	21	6.1	13.6	0.55	2.91	0.5

**Table 4:** Rate constants of photocatalytic decomposition of methyl orange solutions.

Sample	*k*, 10^−3^ min^−1^	*R* ^2^	Sample	*k*, 10^−3^ min^−1^	*R* ^2^
ZnO	3.3 ± 0.5	0.9976	ZnO:0.03V	1.6 ± 0.6	0.7718
ZnO-GO (10 wt.%)	1.8 ± 0.6	0.7980	ZnO:0.05V	1.6 ± 0.8	0.6572
ZnO-GO (20 wt.%)	2.8 ± 0.5	0.9519	ZnO:0.05V-GO (10 wt.%)	2.1 ± 0.7	0.7440
ZnO-GO (30 wt.%)	3.9 ± 0.5	0.9405	ZnO:0.05V-GO (20 wt.%)	2.9 ± 0.5	0.9434
ZnO:0.01V	1.7 ± 0.6	0.8332	ZnO:0.05V-GO (30 wt.%)	4.3 ± 0.5	0.9972

## Data Availability

Data are contained within the article.
